# Pathophysiological processes underlying hidden hearing loss revealed in *Kcnt1/2* double knockout mice

**DOI:** 10.1111/acel.14243

**Published:** 2024-07-24

**Authors:** Nick M. A. Schubert, Daniël O. J. Reijntjes, Marcel van Tuinen, Sarath Vijayakumar, Timothy A. Jones, Sherri M. Jones, Sonja J. Pyott

**Affiliations:** ^1^ Department of Otorhinolaryngology/Head and Neck Surgery, University Medical Center Groningen University of Groningen Groningen The Netherlands; ^2^ Graduate School of Medical Sciences Research School of Behavioural and Cognitive Neurosciences University of Groningen Groningen The Netherlands; ^3^ The Center for Hearing and Balance, Otolaryngology‐Head and Neck Surgery Johns Hopkins University School of Medicine Baltimore Maryland USA; ^4^ Department of Special Education and Communication Disorders, Barkley Memorial Center University of Nebraska Lincoln Lincoln Nebraska USA

**Keywords:** aging, cochlea, hidden hearing loss, mouse, presbycusis, proteostasis

## Abstract

Presbycusis is a prevalent condition in older adults characterized by the progressive loss of hearing due to age‐related changes in the cochlea, the auditory portion of the inner ear. Many adults also struggle with understanding speech in noise despite having normal auditory thresholds, a condition termed “hidden” hearing loss because it evades standard audiological assessments. Examination of animal models and postmortem human tissue suggests that hidden hearing loss is also associated with age‐related changes in the cochlea and may, therefore, precede overt age‐related hearing loss. Nevertheless, the pathological mechanisms underlying hidden hearing loss are not understood, which hinders the development of diagnostic biomarkers and effective treatments for age‐related hearing loss. To fill these gaps in knowledge, we leveraged a combination of tools, including transcriptomic profiling and morphological and functional assessments, to identify these processes and examine the transition from hidden to overt hearing loss. As a novel approach, we took advantage of a recently characterized model of hidden hearing loss: *Kcnt1/2* double knockout mice. Using this model, we find that even before observable morphological pathology, hidden hearing loss is associated with significant alteration in several processes, notably proteostasis, in the cochlear sensorineural structures, and increased susceptibility to overt hearing loss in response to noise exposure and aging. Our findings provide the first insight into the pathophysiology associated with the earliest and, therefore, most treatable stages of hearing loss and provide critical insight directing future investigation of pharmaceutical strategies to slow and possibly prevent overt age‐related hearing loss.

## Graphical abstract

We used a novel mouse model combined with transcriptomic profiling as well as morphological and functional assessments to investigate the pathophysiology of hidden hearing loss. We found that hidden hearing loss is linked to transcriptomic alterations indicating disruption in various molecular and cellular pathways in the cochlear sensorineural structures. Additionally, hidden hearing loss is associated with increased susceptibility to overt hearing loss in response to noise exposure and aging.

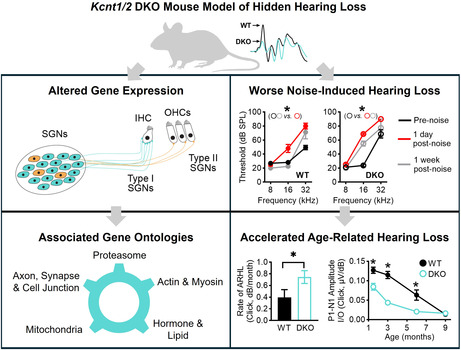

## 1 INTRODUCTION

Normal hearing and auditory communication rely on the transduction, encoding and relaying of acoustic information from the inner ear to the brain. These tasks are the responsibility of specialized sensorineural structures in the cochlea or auditory portion of the inner ear and include the sensory inner hair cells (IHCs), which transduce acoustic stimuli, and the primary auditory neurons or spiral ganglion neurons (SGNs), which relay encoded acoustic signals to the brain. Metabolic structures in the cochlea, including the stria vascularis (SV) and spiral ligament (SL), together maintain cochlear ion homeostasis. Not surprisingly, these structures undergo age‐related changes that contribute to age‐related loss of hearing, which is the most prevalent sensory disorder worldwide (Haile et al., 2021), gives rise to enormous global costs (McDaid et al., 2021), and is associated with the increased risk of frailty, falls, and dementia (Livingston et al., 2020).

Age‐related loss of cochlear function and hearing is progressive and likely preceded by a recently recognized form of “hidden” hearing loss. Hidden hearing loss, so called because it is undetectable by conventional audiological assessment, contributes to deficits in speech discrimination and intelligibility, especially in background noise (Kohrman et al., 2020). Up to 20% of individuals with hearing difficulties are suspected of having hidden hearing loss (Parthasarathy et al., 2020; Spankovich et al., 2018; Tremblay et al., 2015). Examination of animal models and postmortem human tissue have been enormously useful in identifying the morphological pathology underlying hidden hearing loss, which includes loss or disruption of the auditory synapses between the IHCs and SGNs (Hou et al., 2022; Kujawa & Liberman, 2009) and disruption of myelination at the peripheral terminals of the SGNs (Wan & Corfas, 2017). In both models of hidden hearing loss, the observed morphological pathology is consistent with altered neurotransmission and reduced suprathreshold responses to sound that typify hidden hearing loss (Kohrman et al., 2020).

Despite these insights into the pathology associated with hidden hearing loss, the mechanistic processes contributing to functional pathology and preceding morphological pathology remain unknown. Moreover, the possible contribution of the cochlear metabolic structures to the pathology of hidden hearing loss has not been investigated, even though pathology these structures contributes to overt age‐related hearing loss (Yu et al., 2021). Identifying and localizing these mechanisms are an essential first step toward the development of approaches to diagnose and treat hidden hearing loss and prevent the irreversible morphological pathology and progression to overt age‐related hearing loss.

As part of recent work investigating the molecular mechanisms enabling accurate encoding and neurotransmission by SGNs, we identified a phenotype of hidden hearing loss in mice genetically modified to lack Na^+^‐activated potassium (K_Na_) channels, including K_Na_1.1 (KCNT1, SLO2.2 or Slack) and K_Na_1.2 (KCNT2, SLO2.1, or Slick). In mice, transcripts encoding these ion channels have been previously localized to the spiral ganglion neurons (Reijntjes et al., 2019; Shen et al., 2015; Shrestha et al., 2018) with low or no expression found in the sensory hair cells (Liu et al., 2022; Shen et al., 2015) or endocochlear potential generating cells of the stria vascularis (Schubert et al., 2022). Functional expression was confirmed using patch clamp electrophysiology in isolated spiral ganglion neurons (Reijntjes et al., 2019). In vivo investigation revealed that KCNT1/2 double knockout mice (referred to as DKO mice) compared to wild‐type (WT) mice show reduced sound evoked suprathreshold responses, as measured by auditory brainstem response (ABR) wave I amplitudes, without a change in their absolute thresholds (Reijntjes et al., 2019). The phenotype of hidden hearing loss observed in DKO mice occurs without loss of auditory synapses or alterations in the patterns of SGN myelination or nodal architecture (Reijntjes et al., 2019). These findings provide evidence that hidden hearing loss may occur before observable loss of synapses or alterations in myelination although future investigation may identify additional functional and/or morphological deficits. We hypothesized that, like other forms of neurodegenerative disorders, which are marked by impairments that precede and possibly contribute to later neuronal loss (Wilson et al., 2023), hidden hearing loss is also associated with pathophysiological processes that may precede loss or alterations in the spiral ganglion neurons. Therefore, these DKO mice provide an additional animal model to investigate mechanisms associated with hidden hearing loss, including mechanisms that may occur before alterations in spiral ganglion neurons and link the progression from hidden to overt hearing loss.

In this study, we used a broad range of investigative tools to further characterize cochlear dysfunction in DKO mice. We identified the subcellular complexes and their associated processes underlying the phenotype of hidden hearing loss in DKO mice using transcriptomic profiling of inner ear structures. To examine the progression from hidden to overt hearing loss, we examined the effects of noise exposure and aging on cochlear responses and auditory synapse survival in DKO mice using both in vivo functional and in vitro morphological assessments. Our results identify for the first time the pathophysiological processes associated with hidden hearing loss and show that hidden hearing loss is indeed associated with increased susceptibility to overt hearing loss in DKO mice. Ultimately, our work provides new insights into the processes that can be pharmaceutically targeted to prevent, slow, or perhaps reverse hearing loss.

## 2 MATERIALS AND METHODS

### 2.1 Experimental design

Experimental protocols were approved and conducting according to the relevant guidelines and regulations in place at the University Medical Center Groningen (UMCG) and the University of Nebraska Lincoln. DKO mice were bred onto the C57BL/6 background for 12 generations (Martinez‐Espinosa et al., 2015) before being maintained in a breeding colony at the UMCG. To minimize as much as possible the total number of animals used, K_Na_1.1/1.2 (*Kcnt1/2*) DKO mice were investigated to avoid possible compensation for loss of function in single gene KO mice by the other gene and compared to age‐ and sex‐matched C57BL/6 (WT) mice. Previous whole genome scanning was performed to confirm strain identity between C57BL/6 stocks maintained by the UMCG and Jackson Laboratories (Reijntjes et al., 2019).

### 2.2 Cochlear structure isolation, RNA extraction, RNA sequencing, and differential expression analysis

Cochlear structure isolation, RNA extraction, and RNA sequencing were performed as part of a larger study described previously (Reijntjes et al., 2019; Schubert et al., 2022). Substructures from both ears from a single mouse were treated as a single sample. For differential expression analysis, count tables were loaded into the R environment and differential expression analysis was performed with the DESeq2 package. Transcriptional differences between samples WT and DKO samples were compared between cochlear structures and referenced to WT gene expression profiles. Genes with low count numbers were excluded from analysis. Counts were r‐log transformed and visualized with the ggplot2 and pheatmap (http://cran.r‐project.org/web/packages/pheatmap/index.html) packages. Differentially expressed genes (DEGs) were defined by a false discovery rate (FDR) <0.05. DEGs were considered upregulated when log2foldchange (LFC) >0 and downregulated when LFC <0.

### 2.3 Gene ontology enrichment analysis

Gene ontology (GO) enrichment analyses were performed using gProfiler2 package based on previously published protocols (Reimand et al., 2019). Two ranked gene‐lists, consisting of (1) upregulated DEGs of the OC/SGN dataset and (2) downregulated DEGs of the OC/SGN dataset, were uploaded. Statistical significance was determined using g:SCS thresholding within g:Profiler (threshold 0.05). Functional enrichment was performed on the following sources: GO cellular components, biological processes, and molecular function, KEGG pathways, Wiki Pathways and REACTOME. Electronic GO annotations were excluded because of their lower level of evidence.

### 2.4 Aggresome detection

Aggresome detection was performed according to the manufacturer's recommendations (PROTEOSTAT Aggresome Detection Kit, Enzo). Following anesthesia with inhalation of 4% isoflurane, mice were decapitated to remove inner ears. Organs of Corti with the SGNs were isolated from the inner ears and fixed for 45 min at RT in 4% paraformaldehyde (PFA) in phosphate‐buffered saline (PBS). They were then rinsed 3 × 10 min in PBS, incubated 30 min in permeabilization solution on ice, rinsed 3 × 10 min in PBS, incubated 30 min in dual detection reagent (PROTEOSTAT aggresome detection reagent and Hoechst 33342 nuclear stain) covered at RT, rinsed 3 × 10 min in PBS, and then mounted in VECTASHIELD (Vector Laboratories) antifade mounting medium for confocal microscopy. Confocal image z‐stacks of SGNs from identified tonotopic regions (16 kHz) were obtained using a Leica TCS SP8 confocal microscope. Images were taken with the 63× oil immersion objective with a resolution of 1024 × 1024 pixels, a speed between 100 and 400 Hz, and an optical zoom of 1. The z‐step was automatically optimized to the Nyquist sampling frequency. Z‐stacks ranged in thickness to encompass the entire cluster of SGNs. Quantitative image analysis was performed using Imaris v7.6 using the “spots” function to manually mark aggresomes and SGNs. Hoechst staining was excluded from the presented micrographs because it labels all nuclei, including those of the satellite cells, and obscured visualization of the SGNs.

### 2.5 Assessment of cochlear function

Auditory brainstem responses (ABRs) were recorded as described previously (Reijntjes et al., 2019). Mice of either sex were used at the ages indicated. ABR wave I (P1‐N1) amplitude and latency input/output (I/O) regression slopes were calculated for stimuli intensities ranging from 40 to 90 dB SPL in 10 dB steps as described previously (Reijntjes et al., 2019).

### 2.6 Acoustic overexposure

Mice were anesthetized with an intraperitoneal injection of 75 mg/kg ketamine and 1 mg/kg dexmedetomidine. Sham exposed mice were put in an acoustic chamber without presentation of noise. Experimental animals were placed in an acoustic chamber and exposed to a 8–16 kHz noise band at a 100 dB SPL for 120 min using a free field speaker (Visaton DHT 8 S) coupled to an amplifier (Dynavox ET‐100). The tone was generated using dedicated ABR hardware and software (Intelligent Hearing Systems).

### 2.7 Assessment of cochlear morphology

Following anesthesia with either inhalation of 4% isoflurane or with intraperitoneal injection of 75 mg/kg ketamine and 1 mg/kg dexmedetomidine, mice were decapitated to remove inner ears. Isolation and immunostaining of organs of Corti from the inner ears and subsequent microscopy and image analysis were performed as described previously (Reijntjes et al., 2021). Organs of Corti were immunolabeled with a mouse monoclonal (IgG1) anti‐CTBP2 (BD Biosciences, 612044; 1:500) and a rabbit polyclonal anti‐GluA2/3 (Millipore, (AB1506); 1:300). Secondary antibodies included AlexaFluor 488 goat anti‐mouse (IgG1, ThermoFisher, A‐21121) and AlexaFluor 568 goat anti‐rabbit (IgG, ThermoFisher, A‐11011). In some samples, Alexa Fluor 488 phalloidin (ThermoFisher, A12379) was used as described by the manufacturer to identify OHCs. Confocal image z‐stacks of identified tonotopic regions (8, 16, and 32 kHz) of the organ of Corti were acquired as described above for aggresome detection. Z‐stacks ranged in thickness to encompass the entire synaptic pole of the IHCs. Quantitative image analysis was performed using Imaris v7.6 using the “spots” function to detect CTBP2‐labeled presynaptic ribbons and GluA2/3‐labeled postsynaptic glutamate receptor patches. The number of cochlear IHCs was determined by CTBP2‐immunolabeling of manually identified IHC nuclei.

### 2.8 Statistical analyses

All values are presented as the mean ± the standard error of the mean. Data were analyzed using parametric tests when possible. For some data, the assumptions of normality (assessed using the Shapiro–Wilk test) were violated and could not be corrected for by transforming the data. In these cases, non‐parametric Kruskal–Wallis test with post hoc Mann–Whitney *U*‐tests were applied (Figures 3, 5, and 6; Figure S1). *p*‐Values ≤0.05 were considered statistically significant. For linear regression analysis of longitudinal data (Figure 6b), covariates included in the model were genotype, age, stimulus (pure tones or clicks) and the interaction between age and stimulus. For all analyses, thresholds ≥90 dB SPL were scored as 90 dB SPL. These upper values were excluded from linear regression analyses since they do not represent the true numerical absolute threshold. Statistical analyses were performed in either GraphPad Prism or R Statistical Software.

## 3 RESULTS

### 3.1 Abundant changes in gene expression, notably in genes enriched in type I SGNs, are observed in the cochlear sensorineural structures of DKO mice

**FIGURE 1:**
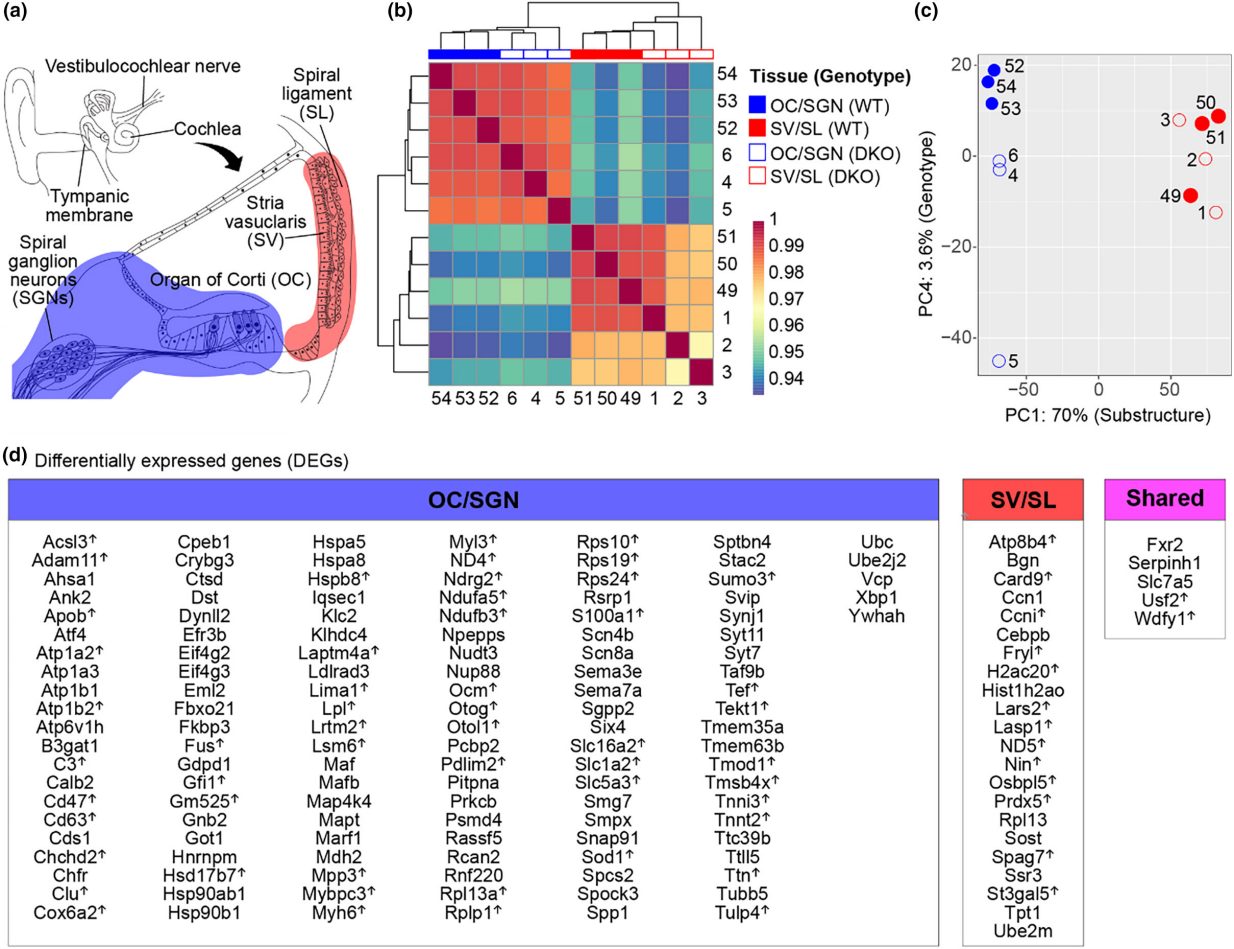
DKO mice show greater transcriptomic changes in the sensorineural compared to metabolic structures of the cochlea. (a) Schematic of the inner ear illustrates the cochlea and cochlear structures, including the sensorineural structures (organ of Corti and spiral ganglion neurons, OC/SGN, blue) and metabolic structures (stria vascularis and spiral ligament, SV/SL, red), isolated for transcriptomic analysis. (b) Correlation heat map with hierarchical clustering analysis shows distinct clustering by structure and genotype (WT and DKO). (c) Principal component analysis (PCA) shows the largest variance is attributed to differences in cochlear structure (PC1). Additional variance among the cochlear sensorineural structures is attributed to genotype (PC4). (d) Differential expression analysis identifies both up‐ and downregulated genes in the cochlear sensorineural (OC/SGN) and metabolic (SV/SL) structures, some of which are shared.

Our previous examination of DKO mice revealed a phenotype of hidden hearing loss—reduced ABR wave I amplitudes despite normal absolute thresholds—without morphological loss or disruption of the sensorineural structures or auditory synapses. To localize and identify the processes underlying the pathophysiology of hidden hearing loss in DKO mice, we used RNA sequencing to examine the transcriptional differences in the sensorineural (organ of Corti and spiral ganglion neurons, OC/SGN) and metabolic (stria vascularis and spiral ligament, SV/SL) structures isolated from the cochleae of WT (*N* = 3) and DKO (*N* = 3) mice (Figure 1a).

We first examined transcriptional differences between structure and genotype by examining sample clustering. Heat map analysis showed distinct clustering by both structure and genotype (Figure 1b). Principal component (PC) analysis (PCA) indicated that the most variation (PC1) was determined by transcriptional differences between structures (70%; Figure 1c). The variation determined by differences in genotype (PC4) accounted for a much smaller fraction (3.6%) of the total variation observed between all samples. Based on PC4, one sample (OC/SGN/DKO5) clustered away from the other five OC/SGN samples, but, given the small amount of variation determined by PC4, this difference is negligible.

Differential expression analysis identified 136 differentially expressed genes (DEGs) between DKO and WT mice in the sensorineural structures (OC/SGN) compared to 27 DEGs in the metabolic structures (SV/SL; Figure 1d, Table S1). The majority of the DEGs in the OC/SGN (81/136 or 60%) were downregulated in DKO compared to WT mice (Figure 1d). In contrast, the majority of DEGs in the SV/SL (15/27 or 56%) were upregulated in DKO compared to WT mice. Only five DEGs were shared between cochlear structures. The fivefold greater number of DEGs observed in the OC/SGN compared to SV/SL suggests that the molecular changes underlying the pathophysiology of hidden hearing loss observed in DKO mice are localized primarily to the sensorineural rather than metabolic structures of the cochlea.

DEGs involved with diverse functions were identified when examining the transcriptional differences between DKO and WT mice in the sensorineural structures (OC/SGN). The three most upregulated genes are *Otol1*, which is expressed in cochlear interphalangeal cells and the border cells (Mulry & Parham, 2020); *Apob*, which is important for low density lipoprotein metabolism; and *Tekt1*, which is expressed in postnatal hair cells (Shen et al., 2015) (Figure 1d, Table S1). The three most downregulated genes are *Slc7a5*, which encodes a neutral amino acid transporter involved in regulation of potassium channel function and hormone transport; *Stac2*, which encodes a member of the STAC family of proteins known to regulate calcium channel function; and *Crybg3*, involved in carbohydrate binding. Other notable DEGs identified in the sensorineural structures include multiple ATPase‐encoding genes, which are either significantly downregulated (e.g., *Atp1a3*, *Atp1b1*, and *Atp6v1h*) or upregulated (e.g., *Atp1a2* and *Atp1b2*). Expression of chaperone heat shock proteins (e.g., *Hspa5*, *Hspa8*, *Hspa90ab1*, and *Hspa90b1*) is mainly downregulated.

When examining the transcriptional changes between DKO and WT mice in the metabolic structures (SV/SL), the three most upregulated genes are *Card9*, *Lars2*, and *H2ac20*, which are involved in cell apoptosis, mitochondrial function, and nucleosome structure, respectively (Figure 1d, Table S2). The three most downregulated genes, *Cebpb*, *Sost*, and *Ccn1*, regulate genes involved in immune and inflammatory responses, bone morphogenesis and transcription factor binding, and cell proliferation and adhesion, respectively (Figure 1d, Table S2).

**FIGURE 2:**
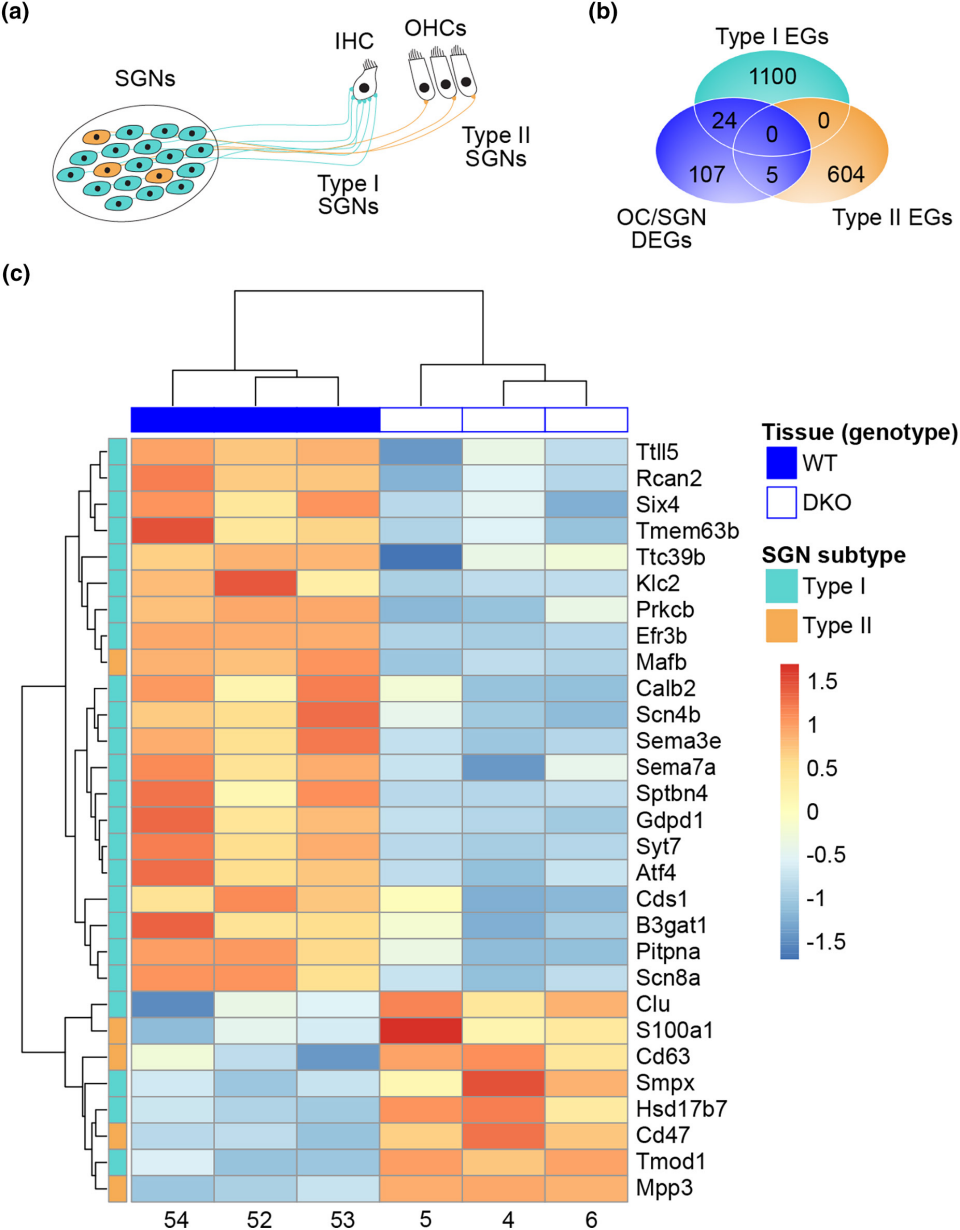
DKO mice show differential expression of genes specifically enriched in type I SGNs. (a) Schematic illustrates the synaptic connectivity between the type I (cyan) and type II (yellow) spiral ganglion neurons (SGNs) and inner and outer hair cells (IHCs and OHCs). (b) Venn diagram shows the overlap in genes differentially expressed in the cochlear sensorineural structures (organ of Corti and spiral ganglion neuron, OC/SGN, differentially expressed genes, DEGs) in DKO mice (blue) compared to previously identified enriched genes (EG) in either the type I (cyan) or type II (yellow) SGNs. (c) Heat map analysis of standardized r‐log transformed expression levels of the overlapping genes show distinct clustering by genotype and SGN subtype.

Previous in vitro investigation identified K_Na_ currents in approximately half of isolated type I SGNs (Reijntjes et al., 2019), which synapse in vivo with the IHCs (Figure 2a). Therefore, we expected that genetic deletion of K_Na_ channels would exert the greatest effect on type I SGNs and, consequently, that DEGs identified in the cochlear sensorineural (OC/SGN) structures of DKO mice would show greater overlap with genes enriched in type I compared to type II SGNs (using the gene set identified in Shrestha et al., 2018). Indeed, of the 136 DEGs in the OC/SGN from DKO mice, 24 (18%) are enriched in the type I SGNs whereas only 5 (4%) were enriched in the type II SGNs. Most of the type I‐specific DEGs (20 out of 24) are downregulated whereas the majority of type II‐specific DEGs (4 out of 5) are upregulated (Figure 2b). The type I SGNs can be further divided into three genetically distinct subtypes (Shrestha et al., 2018). Most of the type I‐specific DEGs were expressed equally abundantly among the three subtypes. Three exceptions included *Calb2*, which is reportedly enriched in type Ia/b SGNs; *B3gat1*, which is enriched in type Ia SGNs; and *Smpx*, which is enriched in type Ib/c SGNs (Figure 2c). Thus, transcriptional changes in the sensorineural structures in DKO mice were greater in type I SGN‐ compared to type II SGN‐specific genes even accounting for the reduced number of type II SGN‐specific genes. Transcriptional changes were, however, not specific to a particular subtype of type I SGNs.

### 3.2 Changes in gene expression are associated with several mechanisms, including altered proteostasis, aging, and deafness, in the cochlear sensorineural structures of DKO mice

**FIGURE 3:**
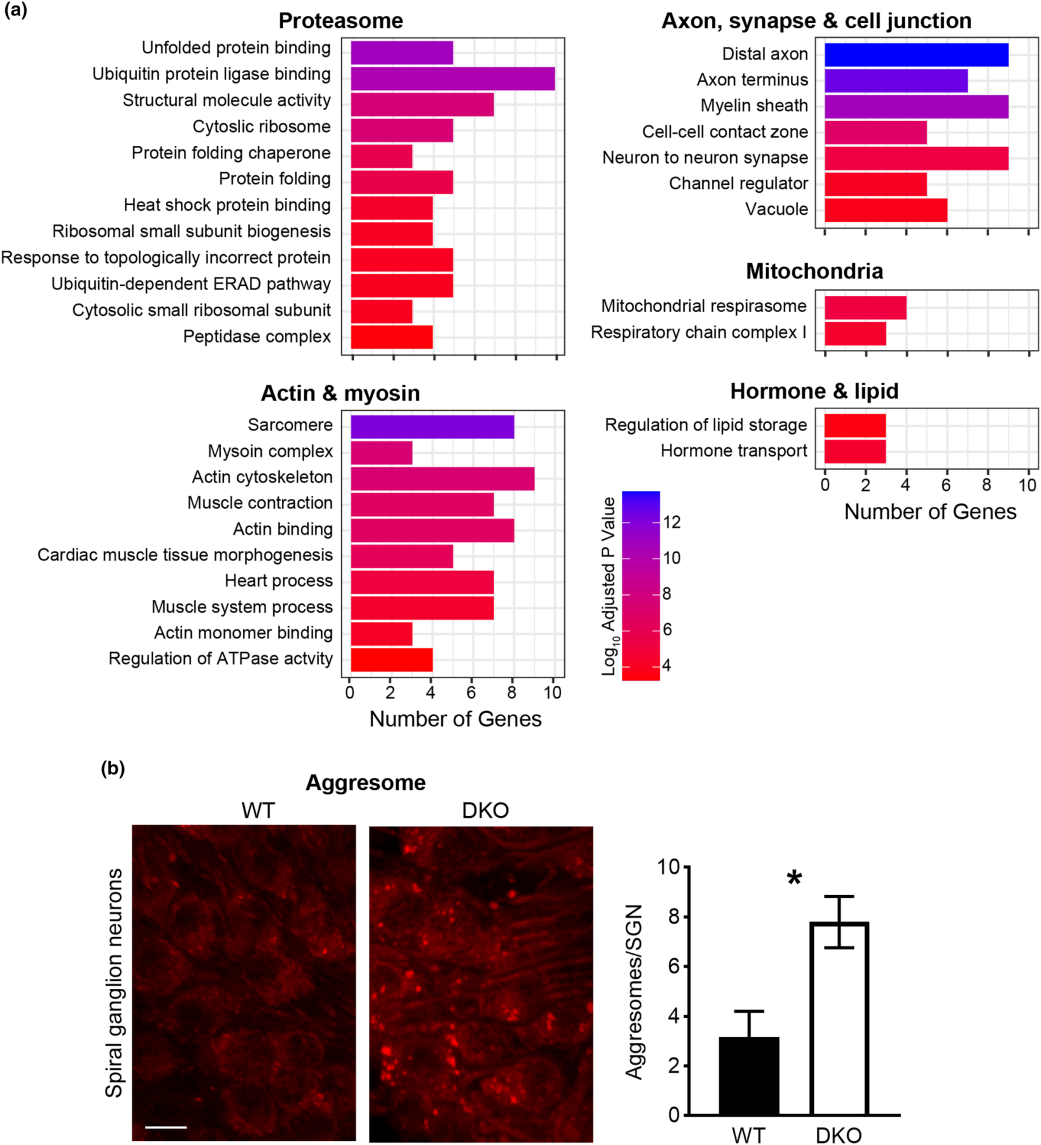
DKO mice show changes in gene expression associated with several mechanisms, including altered proteostasis, in the cochlear sensorineural structures of DKO mice. (a) Gene ontology (GO) enrichment analysis identifies several cellular components, molecular functions, and biological processes that are differentially enriched in the cochlear sensorineural structures (organ of Corti and spiral ganglion neurons, OC/SGN) isolated from DKO mice. GO terms were categorized into five main categories: (1) Proteasome, (2) Actin and myosin, (3) axon, synapse, and cell junction, (4) mitochondria, and (5) hormone and lipid. For many categories, different terms were identified by the same gene list. In this case, only the term with the most significant *p* value is listed. Only terms identified by ≥3 genes are listed. Complete lists are available in Table S3. (b) Z‐projections of PROTEOSTAT‐labeled SGNs isolated from 6‐week‐old WT and DKO mice reveals the greater abundance of aggresomes (red aggregates, left panel) in SGNs from DKO compared to WT mice (right panel). Scale bar equals 10 μm. Significant difference is indicated with an asterisk (*).

To further profile the genes significantly differentially expressed in the cochlear sensorineural (OC/SGN) and metabolic (SV/SL) structures of DKO mice, we performed gene ontology (GO) enrichment analysis. The only enriched GO terms that were identified in the metabolic structures of DKO mice were associated with the extracellular matrix (GO:0050840, GO:0060348, GO:0062023) and were downregulated. Based on separate analysis of the up‐ and downregulated genes, we identified 75 cellular components, molecular functions, and biological processes that are significantly enriched in the set of genes differentially expressed in the cochlear sensorineural structures of DKO mice (g:SCS threshold <0.05; Table S3). The identified GO terms could be broadly categorized into components, functions, and processes relating to the following five categories: (1) proteasome, (2) actin and myosin, (3) axon, synapse, and cell junction, (4) mitochondria, and (5) hormone and lipid (Figure 3a).

Among these five categories, the proteasome category was especially striking because impaired proteasome function has been implicated in many chronic neurodegenerative diseases (Ruz et al., 2020) although not specifically hidden or overt hearing loss. Altered proteasome function was implicated by changes in gene expression across four key subcategories relating to gene transcription (e.g., *Atf4*, *Taf9b*, and *Xbp1*), protein synthesis (e.g., ribosomal proteins, including *Rplp1*, *Rpl13a*, *Rps10*, *Rps19*, and *Rps24* and pre‐mRNA splicing, including *Lsm6*), protein folding (e.g., *Ahsa1*, *Hspa5*, *Hspa8*, *Hsp90ab1*, *Hsp90b1*, and *Serpinh1*), protein trafficking (e.g., *Spcs2*), protein ubiquitination (e.g., *Psmd4*, *Ube2j2*, and *Ubc*), and protein degradation (via peptidase activity, e.g., *Svip* and *Vcp*).

Within the actin and myosin category, all subcategories contained at least two genes encoding proteins related to actin‐myosin interactions (e.g., *Lima1*, *Myl3*, *Myh6*, *Tmod1*, *Tmsb4x*, *Tnni3*, *Tnnt2*, and *Ttn*). The proteins encoded by these genes are enriched in striated muscle and, therefore, result in the enrichment of cardiac and skeletal muscle‐annotated GO terms. Importantly, many of these genes have been previously identified in the sensorineural structures of the cochlea, including *Myl3* in mouse IHCs (Shen et al., 2015) and *Tmod1*, *Tnni3*, *Tnnt2*, and *Ttn* in mouse SGNs (Shen et al., 2015; Shrestha et al., 2018). Of the remaining categories, the three subcategories within the axon, synapse, and cell junction category were identified by genes relating to axon structure (e.g., *Atp1a3*, *Calb2*, *Cpeb1*, *Prkcb*, *Syt7*, and *Syt11*), myelination (e.g., *Pitpna* and *Vcp*), cell–cell contact zones (e.g., *Ank2* and *Ywhah*), synapse structure (e.g., *Ank2*, *Atp1a3*, *Cpeb1*, *Dst*, *Dynll2*, *Mapt*, and *Syt11*), and regulation of channel activity (e.g., *Gnb2*, *Prkcb*, and *Scn4b*). Terms within the mitochondria subcategories were identified by genes encoding proteins important for mitochondrial electron transport (e.g., *Cox6a2*, *Nd4*, *Ndufa5*, and *Ndufb3*). Finally, terms within the hormone and lipid subcategories were identified by genes important for the regulation of hormone transport (e.g., *Slc7a5*) and lipid storage (e.g., *Apob* and *Lpl*).

As a first step to validate transcriptomic changes identifying altered proteasome function in DKO mice, we examined aggresome accumulation in organs of Corti with SGNs isolated from 6‐week‐old WT and DKO mice (Figure 3b). Aggresomes are inclusion bodies of sequestered proteins that form when the proteasomal machinery is overwhelmed by misfolded and aggregated proteins. When fluorescently labeling for the denatured protein cargo present within aggresomes, we observed perinuclear aggresomes in the SGNs. The number of aggresomes per SGN was greater in DKO (7.8 ± 1.0, *N* = 3) compared to WT (3.2 ± 1.0, *N* = 3) mice.

**FIGURE 4:**
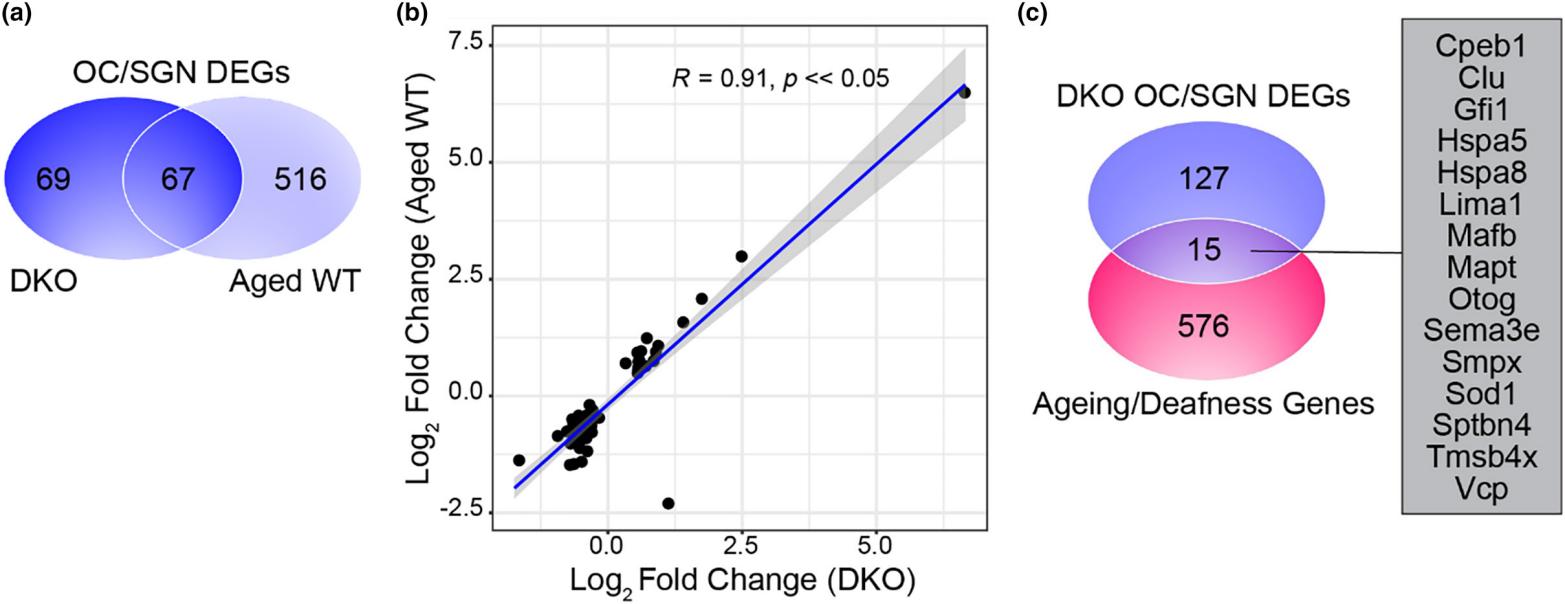
DKO mice show changes in gene expression associated with aging and deafness in the cochlear sensorineural structures of DKO mice (a) Venn diagram shows the overlap in differentially expressed genes (DEGs) in the cochlear sensorineural structures (OC/SGN) in DKO mice (blue) compared to previously identified DEGs identified in the cochlear sensorineural structures of aged (two‐year‐old) mice with overt hearing loss (Aged WT, light blue). (b) There is a significant positive correlation in the fold changes observed in these overlapping DEGs. (c) Venn diagram shows the overlap in differentially expressed genes in the cochlear sensorineural structures (OC/SGN DEGs) in DKO mice (blue) compared to genes previously associated with aging and deafness (purple).

To further characterize the genes associated with the hidden hearing loss phenotype observed in DKO mice, we examined their overlap with genes previously associated with aging and deafness in (WT) mice (Figure 4). First, we investigated the overlap in transcriptional changes in the sensorineural structures (OC/SGN) observed in DKO with hidden hearing loss to the transcriptional changes previously identified in aged (2‐year‐old) mice with overt hearing loss (Schubert et al., 2022). Importantly, transcriptomes from all three groups of mice (i.e., 6‐week‐old WT mice, 6‐week‐old DKO mice, and 2‐year‐old WT mice) were obtained as part of a single, larger RNA sequencing collection, thereby enabling comparison. Approximately half of the genes differentially expressed in DKO mice with hidden hearing loss were also differentially expressed in mice with age‐related, overt hearing loss (*N* = 67/139 or 49%; Figure 4a). There was high correlation between the fold changes in these overlapping DEGs (Pearson's correlation, R = 0.91, *p*‐value <<0.05, Figure 3b). The only gene (1/67) that deviated from this correlation (and was upregulated in DKO but downregulated in aged WT mice) was *Ocm*, which encodes the Ca^2+^‐binding proteins oncomodulin and is enriched in the cochlear outer hair cells (Climer et al., 2019). Next, we investigated the overlap in transcriptional changes observed in the cochlear sensorineural structures from DKO mice with genes previously associated with (1) longevity and/or aging in mice and humans (the GeneAge database in the Human Ageing Genomic Resources); (2) human cellular senescence (the CellAge database in the Human Ageing Genomic Resources); and (3) deafness in mice and humans via the Hereditary Hearing Loss Homepage [accessed October 13, 2021], many of which have been linked to age‐related hearing loss (Boucher et al., 2020; Lewis et al., 2018). A total of 15 genes were identified: *Cpeb1*, *Clu*, *Gfi1*, *Hspa5*, *Hspa8*, *Lima1*, *Mafb*, *Mapt*, *Otog*, *Sema3e*, *Smpx*, *Sod1*, *Sptbn4*, *Tmsb4x*, and *Vcp* (Figure 4c).

### 3.3 Greater susceptibility to noise‐induced and age‐related cochlear pathology is observed in DKO mice

**FIGURE 5:**
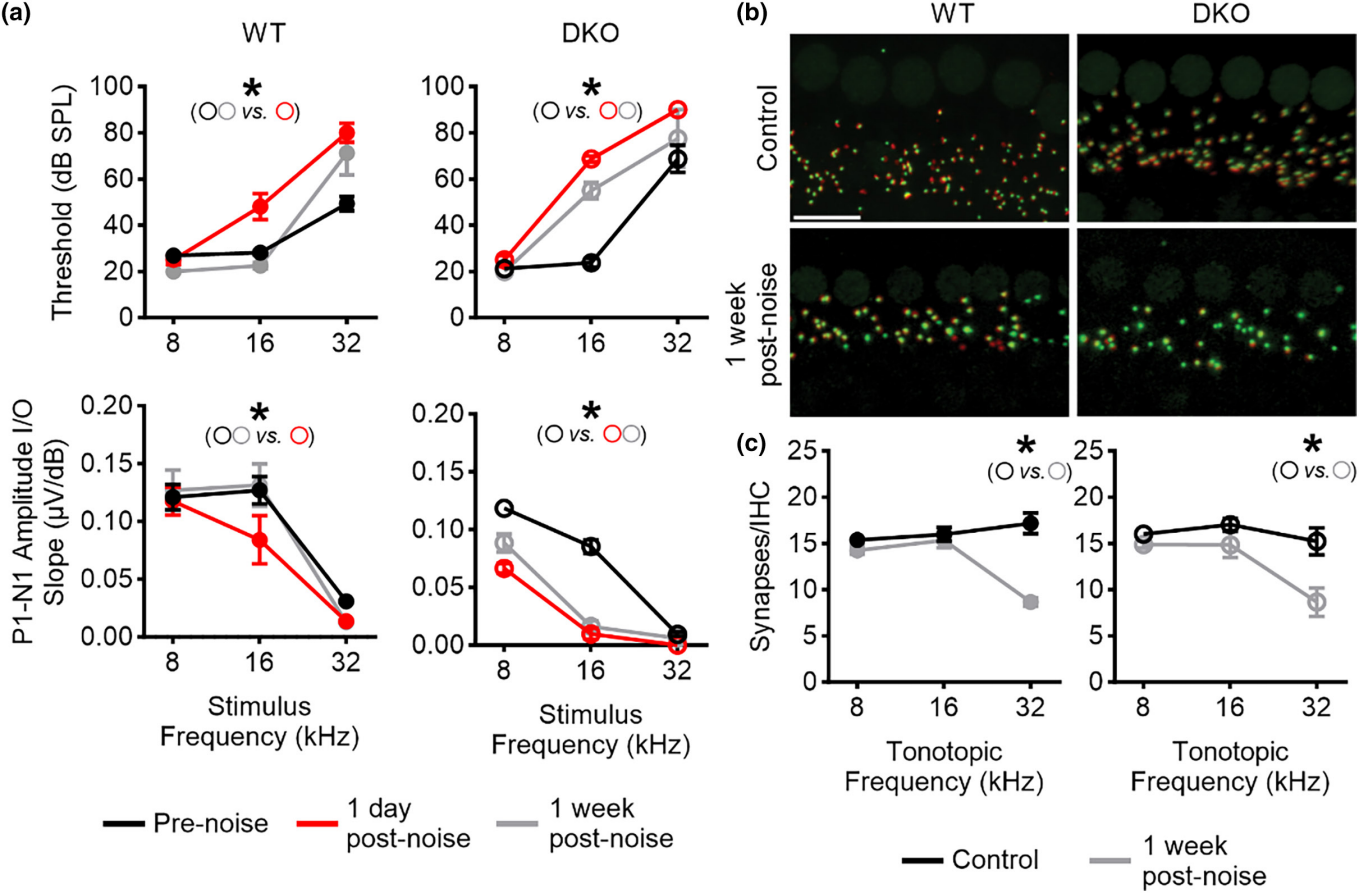
DKO mice show reduced recovery of cochlear function but no differences in auditory synapse loss following noise exposure. (a) Auditory brainstem response (ABR) wave I absolute thresholds (upper panels) and wave I amplitude input/output (I/O) responses (lower panels) before (black line) and 1 day (red line) and 1 week (grey line) after noise exposure are shown as a function of stimulus frequency for both WT (left panels) and DKO (right panels) mice. (b) Z‐projections of the organs of Corti from WT (left panels) and DKO (right panels) mice show CTBP2‐immunostained presynaptic ribbons (green) and GluA2‐immunolabled postsynaptic glutamate receptors (red) in control mice (upper panels) and mice 1 week after noise exposure (lower panels). 32 kHz region is shown. Scale bar equals 10 μm. (c) Synapses (paired presynaptic ribbons and postsynaptic glutamate receptor patches) per inner hair cell (IHC) in control mice (black line) and mice 1 week after noise exposure (grey line) are shown as a function of tonotopic region for WT (filled circles, left panel) and DKO (open circles, right panel) mice. Significant differences are indicated with an asterisk (*).

**FIGURE 6:**
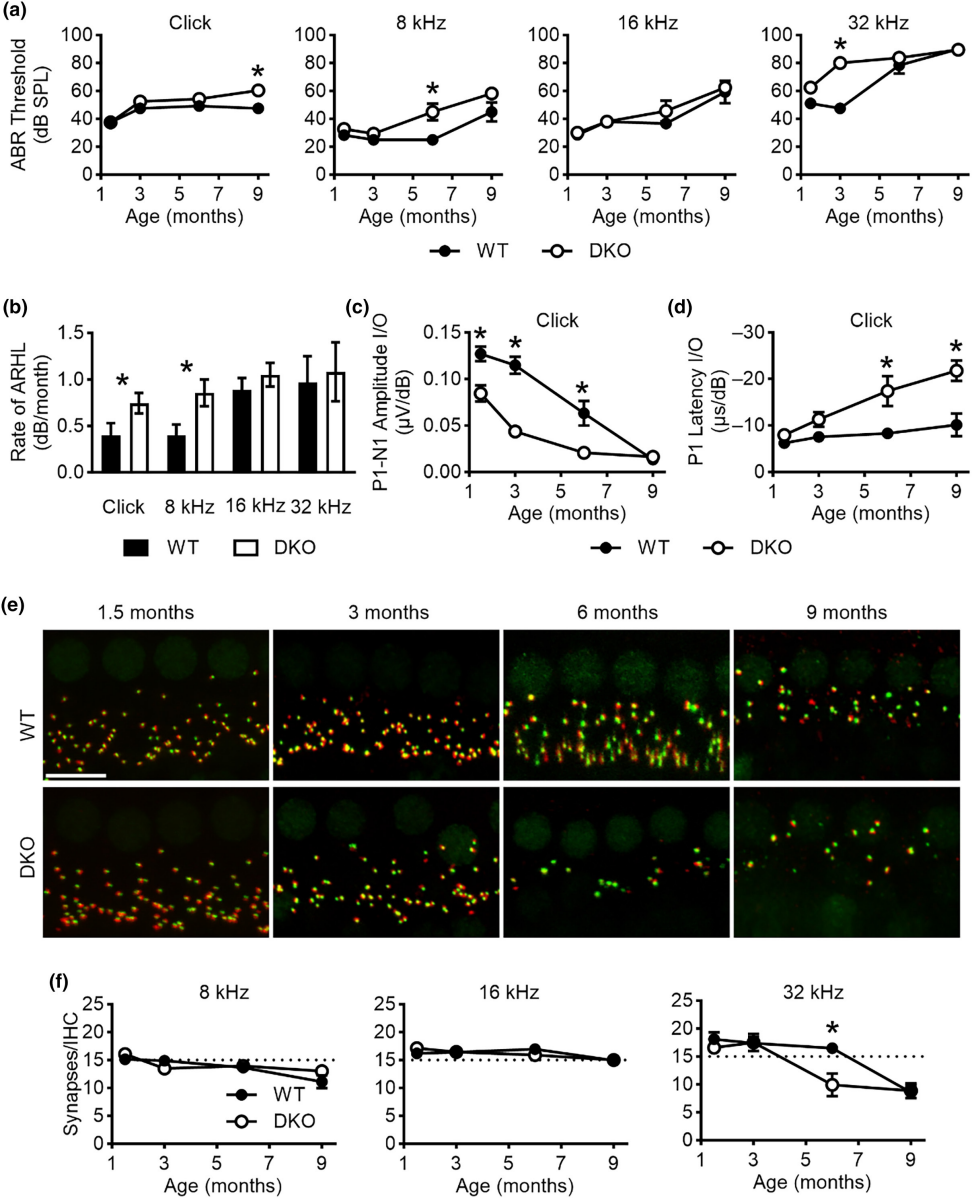
DKO mice show accelerated age‐related loss of cochlear function and auditory synapses. (a) Auditory brainstem response (ABR) wave I absolute thresholds are shown as a function of age for WT (filled circles) and DKO (open circles) mice for the indicated auditory stimuli. (b) The rates of age‐related hearing loss (ARHL) are shown as a function of auditory stimuli for WT (filled bars) and DKO mice (open bars). (c) ABR wave I amplitude input/output (I/O) responses are shown as a function of age for WT (filled circles) and DKO mice (open circles) for click stimuli. (d) ABR wave I latency I/O responses are shown as a function of age for WT (filled circles) and DKO mice (open circles) for click stimuli. (e) Z‐projections of the organs of Corti from WT (upper panels) and DKO (lower panels) mice show CTBP2‐immunostained presynaptic ribbons (green) and GluA2‐immunolabled postsynaptic glutamate receptors (red) at the ages indicated. 32 kHz region is shown. Scale bar equals 10 μm. (f) Synapses (paired presynaptic ribbons and postsynaptic glutamate receptor patches) per IHC are shown as a function of age for WT (filled circles) and DKO mice (open circles) for the indicated tonotopic regions. Significant differences are indicated with an asterisk (*).

Transcriptomic analyses revealed changes in various cellular components, molecular functions, and biological processes within the cochlear sensorineural structures of DKO compared to WT mice (Figures 3 and 4). We suspected these pathophysiological processes associated with hidden hearing loss would also contribute to the increased susceptibility to overt hearing loss in DKO mice. To test this hypothesis and examine whether hidden hearing loss is a precursor of overt hearing loss, we assessed the susceptibility of DKO mice to noise‐induced (Figure 5) and age‐related cochlear pathology (Figure 6).

To test the susceptibility of DKO mice to noise‐induced cochlear pathology (Figure 5a), we assessed cochlear function using auditory brainstem responses (ABRs) to determine ABR wave I absolute thresholds and the growth of the ABR wave I amplitude in response to stimulus intensity (amplitude input/output or I/O responses) before (black line) and then 1 day (red line) and 1 week (grey line) after noise exposure. In these experiments, we selected a noise exposure protocol (8–16 kHz noise band at 100 dB SPL for 120 min) previously shown to maintain the integrity of both the IHCs and OHCs within the frequency range investigated in our study (Fernandez et al., 2020). We examined these two post‐exposure time points to assess the acute response to moderate noise exposure (1 day after noise exposure) as well as recovery from moderate exposure (7 days after noise exposure). These time points were chosen based on previous findings indicating complete recovery by 1‐week post‐noise exposure (Reijntjes et al., 2018). When examining ABR wave I absolute thresholds (upper panels), there was no significant difference in the pre‐ or post‐exposure thresholds at 8 and 32 kHz between WT and DKO mice or within groups of WT and DKO mice at a given frequency. These results are consistent with previous findings using this noise exposure protocol (Fernandez et al., 2020) and also not surprising since frequencies below the noise exposure frequency (i.e., <10 kHz) are not expected to result in hearing loss and since high‐frequency thresholds (i.e., ≥32 kHz) are elevated in this strain of mice (Zheng et al., 1999) and, therefore, mask the effect of noise‐induced hearing loss. In contrast, striking differences in the responses to noise exposure were observed between WT and DKO mice when comparing wave I thresholds measured at 16 kHz. Whereas both WT and DKO showed significant elevations of wave I thresholds acutely after noise exposure (WT: 48.1 ± 5.7 dB SPL, *N* = 8; DKO: 68.8 ± 1.3 dB SPL, *N* = 8), wave I thresholds fully recovered to pre‐noise values in WT mice (pre‐noise: 28.1 ± 0.9 dB SPL, *N* = 8 versus recovery: 22.5 ± 1.4 dB SPL, *N* = 4) but remained elevated in DKO mice (pre‐noise: 23.8 ± 1.8 dB SPL, *N* = 8 vs. recovery: 55.0 ± 3.5 dB SPL, *N* = 4). Similar patterns were observed when examining the increase in wave I amplitude I/O responses (lower panels). There were no significant differences in pre‐ or post‐exposure wave I amplitude I/O responses at 8 and 32 kHz between WT and DKO mice. Wave I amplitude I/O responses were significantly reduced acutely after noise exposure in both WT and DKO mice (WT: 0.084 ± 0.021 μV/dB, *N* = 8; DKO: 0.010 ± 0.005 μV/dB, *N* = 5) and recovered to pre‐noise values in WT mice (pre‐noise: 0.127 ± 0.012 μV/dB, *N* = 8 vs. recovery: 0.131 ± 0.006 μV/dB, *N* = 4) but not DKO mice (pre‐noise: 0.085 ± 0.006 μV/dB, *N* = 8 vs. recovery: 0.016 ± 0.005 μV/dB, *N* = 3). These results indicate that compared to WT mice, DKO mice have impaired recovery of cochlear function following noise exposure.

Since moderate noise exposure resulting in hidden hearing loss has been associated with the structural loss of auditory synapses between the type I SGNs and IHCs (Kujawa & Liberman, 2009), we used immunofluorescence to quantify synapse loss following noise exposure in organs of Corti isolated from WT and DKO mice (Figure 5b). Intact synapses were indicated by colocalization of CTBP2‐positive presynaptic ribbons (green) with GluA2/3‐positive postsynaptic glutamate receptor patches (red). When examining across samples (Figure 5c), there was no significant loss of synapses in the 8 and 16 kHz regions. There was, however, significant yet equivalent loss of synapses in the 32 kHz region in both WT (pre‐noise: 17.2 ± 1.1 synapses, *N* = 8 vs. recovery: 8.7 ± 0.4, *N* = 10) and DKO mice (pre‐noise: 15.2 ± 1.5 synapses, *N* = 8 vs. recovery: 8.6 ± 01.5, *N* = 8).Together with the results from functional assessments, these results indicate that the loss of cochlear function in the 16 kHz region in DKO mice following noise exposure is not due to structural loss of the auditory synapses.

We also examined the susceptibility of DKO mice to age‐related cochlear pathology (Figure 6). We compared ABR wave I absolute thresholds at 1.5, 3, 6, and 9 months of age in response to click stimuli and pure tones at 8, 16, and 32 kHz (Figure 6a). DKO and WT mice showed similar wave I thresholds in response to clicks and pure tones at 1.5 months of age, consistent with the absence of overt hearing loss in young DKO mice reported previously (Reijntjes et al., 2019). As expected for this background strain (Zheng et al., 1999), both DKO and WT mice showed age‐related increases in wave I thresholds progressing from high to low frequencies. Even with this strain‐specific age‐related hearing loss, age‐related hearing loss was accelerated in DKO compared to WT mice. Specifically, DKO compared to WT mice showed significantly elevated wave I thresholds in response to high frequency tones (i.e., 32 kHz) already at 3 months of age (WT: 47.5 ± 1.4 dB SPL, *N* = 8 vs. DKO: 80.0 ± 0.0 dB SPL, *N* = 6) that progressed to low frequency tones (i.e., 8 kHz) by 6 months of age (WT: 45.0 ± 6.7 dB SPL, *N* = 6 vs. DKO: 58.2 ± 4.1 dB SPL, *N* = 14) and were detectable in response to click stimuli at 9 months of age (WT: 47.5 ± 1.7 dB SPL, *N* = 6 vs. DKO: 60.4 ± 2.9 dB SPL, *N* = 14). Linear regression analysis to calculate the rate of hearing loss (rate of wave I threshold elevation in dB per month; Figure 6b) showed that DKO mice have significantly increased rates of hearing loss in response to click stimuli (WT: 0.40 ± 0.13 dB/month, *N* = 6 vs. DKO: 0.74 ± 0.11 dB/month, *N* = 14) and 8 kHz pure tones (WT: 0.39 ± 0.14 dB/month, *N* = 6 vs. DKO: 0.86 ± 0.141 dB/month, *N* = 14). Thus, the rate of age‐related hearing loss is almost twice as fast in DKO compared to WT mice. Although DKO compared to WT mice showed significantly larger wave I thresholds at 32 kHz at 3 months of age, statistically significant differences in the rate of age‐related hearing loss at higher frequencies (i.e., 16 and 32 kHz) were not detected due to the early, steep rise in threshold elevations in both genotypes.

We also examined ABR wave I amplitude (Figure 6c) and latency (Figure 6d) I/O responses at 1.5, 3, 6, and 9 months of age in response to click stimuli. As expected from our previous findings (Reijntjes et al., 2019), at 1.5 months of age, wave I amplitude I/O responses were significantly reduced in DKO (0.08 ± 0.01 μv/dB, *N* = 31) compared to WT (0.13 ± 0.01 μv/dB, *N* = 30) mice in response to clicks, consistent with hidden hearing loss in DKO mice. With increasing age, both DKO and WT mice showed decreasing wave I amplitude I/O responses, but responses in DKO mice were still significantly reduced at 3 (WT: 0.12 ± 0.01 μv/dB, *N* = 8 vs. DKO: 0.04 ± 0.01 μv/dB, *N* = 8) and 6 (WT: 0.06 ± 0.001 μv/dB, *N* = 6 vs. DKO: 0.02 ± 0.003 μv/dB, *N* = 4) months of age. By 9 months of age, wave I amplitude I/O responses showed similar reductions in both DKO and WT mice. When examining ABR wave I latency I/O responses, no significant differences were observed between DKO and WT mice at 1.5 weeks or 3 months of age, consistent with previous findings (Reijntjes et al., 2019). DKO compared to WT mice showed significantly slower wave I latency responses at both 6 (WT: −8.3 ± 0.9 μs/dB, *N* = 7 versus DKO: −17.4 ± 3.1 μs/dB, *N* = 5) and 9 (WT: −10.2 ± 2.4 μs/dB, *N* = 5 versus DKO: −21.8 ± 6.4 μs/dB, *N* = 9) months of age. These results indicate that age‐related loss of cochlear function is accelerated in DKO compared to WT mice.

Age‐related hearing loss has been associated with the structural loss of auditory synapses between the type I SGNs and IHCs. Therefore, we used immunofluorescence to quantify synapse loss at 1.5, 3, 6, and 9 months of age in organs of Corti isolated from WT and DKO mice (Figure 6e). Intact synapses were again indicated by colocalization of CTBP2‐positive presynaptic ribbons (green) with GluA2/3‐positive postsynaptic glutamate receptor patches (red). When examining across samples (Figure 6f), there was no significant loss of synapses in the 8 and 16 kHz regions either across ages or between WT and DKO mice. There was, however, significant loss of synapses in the 32 kHz region at 6 months of age in DKO (9.9 ± 2.0 synapses, *N* = 6) compared to WT (16.5 ± 0.6 synapses, *N* = 7) mice. By 9 months of age, both DKO and WT mice showed comparable reductions in the number of synapses (approximately 50%) in the 32 kHz region. When examining age‐related loss of IHCs and OHCs, we found no significant differences between genotypes at each age examined (Figure S1). In both WT and DKO mice, IHC density remained stable up to 9 months of age across tonotopic regions, including the highest tonotopic region at 32 kHz (WT: 10.4 ± 0.5 IHC/100 μm, *N* = 6 compared to DKO: 11.0 ± 0.4 IHC/100 μm, *N* = 8; Figure S1a). OHC density remained consistent across tonotopic regions up to 9 months of age, except for the highest tonotopic region at 32 kHz, where there was a notable loss of OHCs between 6 (WT: 33.9 ± 2.7 OHC/100 μm, *N* = 3 compared to DKO: 35.5 ± 3.9 OHC/100 μm, *N* = 3; Figure S1b) and 9 months of age, when OHCs could only be found in one of the three animals investigated per genotype (WT: 0.2 OHC/100 μm, *N* = 3 compared to DKO: 6.9 OHC/100 μm, *N* = 3; Figure S1b). Together with the results from functional assessments, these results indicate that age‐related loss of cochlear function precedes structural loss of the auditory synapses in both WT and DKO mice. The eventual loss of auditory synapses in high‐frequency cochlear regions occurs earlier in DKO compared to WT mice. There is no difference in the age‐related loss of IHCs and OHCs.

## 4 DISCUSSION

Current models of hidden hearing loss indicate pathology of the auditory neurons (SGNs) and their synapses with the inner hair cells but do not per se localize or identify the underlying pathological processes. The function of the auditory neurons and sensory hair cells is critically dependent upon the integrity of the molecular and cellular signaling complex contained in the cochlear sensorineural structures as well as the cochlear metabolic structures that maintain the ionic composition of the endolymph. We observed transcriptomic changes predominantly in the cochlear sensorineural compared to metabolic structures of DKO mice and predominantly in the type I compared to type II SGNs. These findings are consistent with pathology localized to the subtype of auditory neurons responsible for encoding and relaying acoustic information necessary for perception. Our work nevertheless indicates that there are abundant transcriptional changes in genes that are not specifically enriched in the SGNs and in genes associated with subcellular structures and macromolecular complexes that are not specifically sensorineural. This finding motivates continued work investigating the contribution of non‐neuronal cells in hidden hearing loss.

When examining the pathophysiological mechanisms associated with hidden hearing loss in these DKO mice, we identified processes related to altered proteostasis, aging, and deafness within the cochlear sensorineural structures in DKO mice. Alterations in proteostasis were identified by changes in the expression of genes involved in protein synthesis, folding, trafficking, ubiquitination, and degradation and further suggested by the increased abundance of aggresomes in the SGNs from DKO compared to WT mice. Perturbations of proteostasis resulting in proteotoxicity are key drivers of various neurodegenerative disorders (Ruz et al., 2020) and associated with aging (Pras & Nollen, 2021). More recent findings link proteotoxicity to various forms of hearing loss (Pouyo et al., 2021). Aggresomes specifically play an important role in neurodegenerative disorders (Olzmann et al., 2008). Although aggresome accumulation has not been examined in the context of neurodegeneration associated with age‐related pathology of the cochlea, aggresome accumulation in the developing SGNs has been associated with impaired cochlear development and deafness in genetically disrupted proteostasis (Freeman et al., 2019). Our findings linking proteotoxicity, aging, and deafness to hidden hearing loss are, therefore, not surprising and worthy of further investigation using additional approaches, including, for example, immunofluorescence with antibodies known to bind protein components of aggresomes and transmission electron microscopy to further examine their structure and presence in the SGNs as well as other sensorineural cell types.

We also found that numerous processes related to actin/myosin and a smaller number of processes related to mitochondria as well as hormone transport and lipid storage are altered in the cochlear sensorineural structures in DKO mice. Actin and myosin are required for diverse cellular functions, including muscle contraction, cell motility, cell adhesion, cytokinesis, and organelle transport. Mutations in genes encoding actin, myosin, as well as actin/myosin‐interacting proteins have been linked to hearing loss (Brownstein et al., 2014; Drummond et al., 2012; Libby & Steel, 2000). Investigation of these models suggests that the normal function of these genes serves to protect the hair cell stereocilia from the nearly constant mechanical stress imposed by acoustic stimulation. None of the actin/myosin‐related genes that were differentially expressed in DKO mice have been previously associated with hearing loss, suggesting that altered actin/myosin‐dependent processes identified by GO enrichment analysis in DKO mice may affect structures other than the hair cell stereocilia and/or more dynamically regulated processes. Mitochondria and mitochondrial oxidative stress have long been linked to hearing loss (Böttger & Schacht, 2013). More recent findings link dyslipidemia to sudden sensorineural hearing loss (Li et al., 2021). Although differentially expressed genes associated with hormone transport are expressed in SGNs, including *Slc7a5* and *Syt7* (Shrestha et al., 2018) as well as *Spp1* (Kim et al., 2014), their link to hidden hearing loss is less clear.

To gain insights into the transition from hidden to overt hearing loss, we examined the susceptibility of DKO mice to both noise‐induced and age‐related hearing loss. DKO mice showed greater susceptibility to noise‐induced hearing loss and accelerated age‐related hearing loss compared to WT mice. Most notably, whereas WT mice showed complete recovery of both ABR wave I thresholds and amplitude I/O responses following noise exposure, DKO failed to show any recovery. Previous work using mass spectrometry‐based quantitative proteomics found that noise exposure in mice triggers robust cochlear proteotoxic stress both acutely and during recovery and concluded that protein synthesis is a crucial contributor to threshold recovery (Jongkamonwiwat et al., 2020). These previous findings together with our observation of altered proteostasis in DKO mice suggest that the increased susceptibility noise‐induced hearing loss in DKO mice results specifically from impaired recovery following noise exposure and should be investigated further. In addition to their increased susceptibility to noise‐induced hearing loss, DKO mice also showed accelerated age‐related hearing loss compared to WT mice when examining both ABR wave I absolute thresholds and amplitude I/O responses. The remarkable overlap in transcriptomic changes documented in young DKO mice with hidden hearing loss and old WT mice with overt, age‐related hearing loss suggests that the mechanisms underlying both types of hearing loss are shared. Our findings collectively suggest that hidden hearing loss is a precursor of overt hearing loss.

Our investigation of hidden hearing loss in DKO mice extends previous research, offering further insights into mechanisms linking hidden and overt hearing loss. First, we identified mostly reduced expression of genes previously associated with loss of function mutations linked to overt hearing loss, including *Gfi1*, *Otog*, *Mafb*, *Sema3e*, *Smpx* and *Sptbn4*, suggesting hidden and overt hearing loss share similar genetic mechanisms. Second, we found that *Sod1*, which encodes superoxide dismutase 1 (SOD1), is upregulated in the cochlear sensorineural structures in DKO mice. Notably, previous research has demonstrated that decreased SOD1 expression, which likely exacerbates oxidative stress, increases vulnerability to noise‐induced hearing loss (Ohlemiller et al., 1999) and age‐related hearing loss (McFadden et al., 1999). However, it has also been shown that overexpression of SOD1 does not confer protection against hearing loss (Coling et al., 2003). Thus, altered *Sod1* expression in DKO mice suggests elevated oxidative stress contributes not only to overt hearing loss but also hidden hearing loss and, moreover, that increased *Sod1* expression is insufficient in protecting against hidden and subsequent overt hearing loss. Finally, previous work examining the same line of DKO mice (Lee et al., 2019) attributed the overt hearing loss observed in 5‐month‐old DKO mice with caspase‐mediated apoptotic neurodegeneration based on immunofluorescent detection of activated caspase 3 in SGNs (Lee et al., 2019). In our study, we found no transcriptomic evidence suggesting the activation of caspase or caspase‐mediated apoptosis in the cochlear sensorineural structures of younger (1.5‐month‐old) DKO mice with hidden hearing loss. Thus, we suspect that caspase‐mediated apoptotic pathways are associated with overt hearing loss and may be important in the transition from hidden to overt hearing loss. Future work should aim to substantiate the mechanisms we identified as part of this study with both longitudinal transcriptomic analyses as well as proteomic approaches also in other mouse models of hidden hearing loss.

We also recognize two issues in the experimental design. First, to reduce the total numbers of animals as well as enhance the efficiency and coordination of breeding animals required for investigation, experiments were performed on DKO backcrossed to the C57BL/6J line (Martinez‐Espinosa et al., 2015) and compared to separately bred WT mice originating from the same strain. To exclude as best as possible phenotypic differences that might arise from genetic drift between these two separately breeding lines, we confirmed strain identity between lines using whole genome scanning (Reijntjes et al., 2019). Secondly, in this study, bulk RNA sequencing was used to identify differential gene expression between the cochlear sensorineural and metabolic substructures from WT and DKO. This approach is advantageous for investigations (like this one) focusing on transcriptomic changes affecting the overall gene expression landscape of tissues, rather than alterations specific to individual cells. Additionally, bulk RNA sequencing requires less extensive tissue processing. However, the application of single‐cell RNA sequencing would complement our findings and uncover the cell‐specific transcriptional changes contributing to the observed phenotypic differences between WT and DKO mice.

In conclusion, our findings reveal several pathophysiological processes that are associated with hidden hearing loss and initiated before the structural loss of the auditory synapses (Kujawa & Liberman, 2009) and demyelination of the SGNs (Wan & Corfas, 2017). Our research broadens the spectrum of animal models available for investigating hidden hearing loss, which is crucial for comprehensively understanding the diverse pathophysiological mechanisms underlying this condition in humans (Kohrman et al., 2020). Furthermore, our study contributes to the growing body of evidence implicating potassium and other ion channels in age‐related hearing loss (Bazard et al., 2021; Peixoto Pinheiro et al., 2021), thereby offering potential avenues for drug discovery (Schubert et al., 2022). Moreover, our findings point toward promising pharmaceutical strategies, such as the utilization of proteostasis‐modifying drugs (Lualdi et al., 2020) to treat hearing loss mechanistically.

## AUTHOR CONTRIBUTIONS

Conceptualization, N.M.A.S., D.O.J.R. and S.J.P.; data curation, N.M.A.S., D.O.J.R., S.V., M.v.T. and S.J.P.; formal analysis, N.M.A.S., D.O.J.R., S.V. and M.v.T.; funding acquisition, N.M.A.S. and S.J.P.; investigation, N.M.A.S. and D.O.J.R.; methodology, N.M.A.S., D.O.J.R., S.V. and M.v.T.; project administration, S.J.P.; resources, S.J.P.; software, N.M.A.S. and D.O.J.R.; supervision, T.A.J., S.M.J. and S.J.P.; validation, S.J.P.; visualization, N.M.A.S., D.O.J.R. and S.J.P.; writing—original draft, N.M.A.S. and D.O.J.R.; writing—review and editing, T.A.J., S.M.J., M.v.T. and S.J.P. All authors have read and agreed to the published version of the manuscript.

## FUNDING INFORMATION

This work was supported by funds from the Graduate School of Medical Sciences (MD/PhD scholarship number 16‐59) from the University of Groningen to N.M.A.S. and the Heinsius Houbolt Foundation to S.J.P. Part of the work has been performed at the UMCG Imaging and Microscopy Center (UMIC), which is sponsored by Dutch Research Council (NWO) grants 40‐00506‐98‐9021 and 175‐010‐2009‐023.

## CONFLICT OF INTEREST STATEMENT

The authors declare no competing interests.

## Supporting information


Figure S1.

Table S1.

Table S2.

Table S3.

## Data Availability

All relevant data are included in the manuscript or its Supplementary Information and available from the authors upon request. Transcriptome data are uploaded an available via the University of Maryland Gene Expression Analysis Resource portal: https://umgear.org/p?s=c29f9acf.

## References

[acel14243-bib-0001] Bazard, P. , Frisina, R. D. , Acosta, A. A. , Dasgupta, S. , Bauer, M. A. , Zhu, X. , & Ding, B. (2021). Roles of key ion channels and transport proteins in age‐related hearing loss. International Journal of Molecular Sciences, 22, 6158. 34200434 10.3390/ijms22116158 PMC8201059

[acel14243-bib-0003] Boucher, S. , Tai, F. W. J. , Delmaghani, S. , Lelli, A. , Singh‐Estivalet, A. , Dupont, T. , Niasme‐Grare, M. , Michel, V. , Wolff, N. , Bahloul, A. , Bouyacoub, Y. , Bouccara, D. , Fraysse, B. , Deguine, O. , Collet, L. , Thai‐Van, H. , Ionescu, E. , Kemeny, J.‐L. , Giraudet, F. , … Petit, C. (2020). Ultrarare heterozygous pathogenic variants of genes causing dominant forms of early‐onset deafness underlie severe presbycusis. Proceedings of the National Academy of Sciences of the United States of America, 117, 31278–31289. 33229591 10.1073/pnas.2010782117 PMC7733833

[acel14243-bib-0004] Brownstein, Z. , Abu‐Rayyan, A. , Karfunkel‐Doron, D. , Sirigu, S. , Davidov, B. , Shohat, M. , Frydman, M. , Houdusse, A. , Kanaan, M. , & Avraham, K. B. (2014). Novel myosin mutations for hereditary hearing loss revealed by targeted genomic capture and massively parallel sequencing. European Journal of Human Genetics, 22, 768–775. 24105371 10.1038/ejhg.2013.232 PMC4023209

[acel14243-bib-0002] Böttger, E. C. , & Schacht, J. (2013). The mitochondrion: A perpetrator of acquired hearing loss. Hearing Research, 303, 12–19. 23361190 10.1016/j.heares.2013.01.006 PMC3681877

[acel14243-bib-0005] Climer, L. K. , Cox, A. M. , Reynolds, T. J. , & Simmons, D. D. (2019). Oncomodulin: The enigmatic Parvalbumin protein. Frontiers in Molecular Neuroscience, 12, 235. 31649505 10.3389/fnmol.2019.00235 PMC6794386

[acel14243-bib-0006] Coling, D. E. , Yu, K. C. Y. , Somand, D. , Satar, B. , Bai, U. , Huang, T.‐T. , Seidman, M. D. , Epstein, C. J. , Mhatre, A. N. , & Lalwani, A. K. (2003). Effect of SOD1 overexpression on age‐ and noise‐related hearing loss. Free Radical Biology & Medicine, 34, 873–880. 12654476 10.1016/s0891-5849(02)01439-9

[acel14243-bib-0007] Drummond, M. C. , Belyantseva, I. A. , Friderici, K. H. , & Friedman, T. B. (2012). Actin in hair cells and hearing loss. Hearing Research, 288, 89–99. 22200607 10.1016/j.heares.2011.12.003 PMC3403717

[acel14243-bib-0008] Fernandez, K. A. , Guo, D. , Micucci, S. , De Gruttola, V. , Liberman, M. C. , & Kujawa, S. G. (2020). Noise‐induced cochlear synaptopathy with and without sensory cell loss. Neuroscience, 427, 43–57. 31887361 10.1016/j.neuroscience.2019.11.051 PMC7450393

[acel14243-bib-0009] Freeman, S. , Mateo Sánchez, S. , Pouyo, R. , Van Lerberghe, P.‐B. , Hanon, K. , Thelen, N. , Thiry, M. , Morelli, G. , Van Hees, L. , Laguesse, S. , Chariot, A. , Nguyen, L. , Delacroix, L. , & Malgrange, B. (2019). Proteostasis is essential during cochlear development for neuron survival and hair cell polarity. EMBO Reports, 20, e47097. 31321879 10.15252/embr.201847097 PMC6726910

[acel14243-bib-0010] Haile, L. M. , Kamenov, K. , Briant, P. S. , Orji, A. U. , Steinmetz, J. D. , Abdoli, A. , Abdollahi, M. , Abu‐Gharbieh, E. , Afshin, A. , Ahmed, H. , Rashid, T. A. , Akalu, Y. , Alahdab, F. , Alanezi, F. M. , Alanzi, T. M. , Hamad, H. A. , Ali, L. , Alipour, V. , Al‐Raddadi, R. M. , … Chadha, S. (2021). Hearing loss prevalence and years lived with disability, 1990–2019: Findings from the global burden of disease study 2019. The Lancet, 397, 996–1009. 10.1016/S0140-6736(21)00516-X PMC7960691 33714390

[acel14243-bib-0011] Hou, S. , Zhang, J. , Wu, Y. , Junmin, C. , Yuyu, H. , He, B. , Yang, Y. , Hong, Y. , Chen, J. , Yang, J. , & Li, S. (2022). FGF22 deletion causes hidden hearing loss by affecting the function of inner hair cell ribbon synapses. Frontiers in Molecular Neuroscience, 15, 922665. 35966010 10.3389/fnmol.2022.922665 PMC9366910

[acel14243-bib-0012] Jongkamonwiwat, N. , Ramirez, M. A. , Edassery, S. , Wong, A. C. Y. , Yu, J. , Abbott, T. , Pak, K. , Ryan, A. F. , & Savas, J. N. (2020). Noise exposures causing hearing loss generate Proteotoxic stress and activate the Proteostasis network. Cell Reports, 33, 108431. 33238128 10.1016/j.celrep.2020.108431 PMC7722268

[acel14243-bib-0013] Kim, H. J. , Ryu, J. , Woo, H.‐M. , Cho, S. S. , Sung, M. K. , Kim, S. C. , Park, M.‐H. , Park, T. , & Koo, S. K. (2014). Patterns of gene expression associated with Pten deficiency in the developing inner ear. PLoS One, 9, e97544. 24893171 10.1371/journal.pone.0097544 PMC4043736

[acel14243-bib-0014] Kohrman, D. C. , Wan, G. , Cassinotti, L. , & Corfas, G. (2020). Hidden hearing loss: A disorder with multiple etiologies and mechanisms. Cold Spring Harbor Perspectives in Medicine, 10, a035493. 30617057 10.1101/cshperspect.a035493 PMC6612463

[acel14243-bib-0015] Kujawa, S. G. , & Liberman, M. C. (2009). Adding insult to injury: Cochlear nerve degeneration after “temporary” noise‐induced hearing loss. The Journal of Neuroscience, 29, 14077–14085. 19906956 10.1523/JNEUROSCI.2845-09.2009 PMC2812055

[acel14243-bib-0016] Lee, J. H. , Kang, M. , Park, S. , Perez‐Flores, M. C. , Zhang, X.‐D. , Wang, W. , Gratton, M. A. , Chiamvimonvat, N. , & Yamoah, E. N. (2019). The local translation of KNa in dendritic projections of auditory neurons and the roles of KNa in the transition from hidden to overt hearing loss. Aging (Albany NY), 11, 11541–11564. 31812952 10.18632/aging.102553 PMC6932877

[acel14243-bib-0017] Lewis, M. A. , Nolan, L. S. , Cadge, B. A. , Matthews, L. J. , Schulte, B. A. , Dubno, J. R. , Steel, K. P. , & Dawson, S. J. (2018). Whole exome sequencing in adult‐onset hearing loss reveals a high load of predicted pathogenic variants in known deafness‐associated genes and identifies new candidate genes. BMC Medical Genomics, 11, 77. 30180840 10.1186/s12920-018-0395-1 PMC6123954

[acel14243-bib-0018] Li, X. , Chen, B. , Zhou, X. , Ye, F. , Wang, Y. , & Hu, W. (2021). Identification of dyslipidemia as a risk factor for sudden sensorineural hearing loss: A multicenter case‐control study. Journal of Clinical Laboratory Analysis, 35, e24067. 34674306 10.1002/jcla.24067 PMC8649383

[acel14243-bib-0019] Libby, R. T. , & Steel, K. P. (2000). The roles of unconventional myosins in hearing and deafness. Essays in Biochemistry, 35, 159–174. 12471897 10.1042/bse0350159

[acel14243-bib-0020] Liu, H. , Giffen, K. P. , Chen, L. , Henderson, H. J. , Cao, T. A. , Kozeny, G. A. , Beisel, K. W. , Li, Y. , & He, D. Z. (2022). Molecular and cytological profiling of biological aging of mouse cochlear inner and outer hair cells. Cell Reports, 39, 110665. 35417713 10.1016/j.celrep.2022.110665 PMC9069708

[acel14243-bib-0021] Livingston, G. , Huntley, J. , Sommerlad, A. , Ames, D. , Ballard, C. , Banerjee, S. , Brayne, C. , Burns, A. , Cohen‐Mansfield, J. , Cooper, C. , Costafreda, S. G. , Dias, A. , Fox, N. , Gitlin, L. N. , Howard, R. , Kales, H. C. , Kivimäki, M. , Larson, E. B. , Ogunniyi, A. , … Mukadam, N. (2020). Dementia prevention, intervention, and care: 2020 report of the lancet commission. The Lancet, 396, 413–446. 10.1016/S0140-6736(20)30367-6 PMC7392084 32738937

[acel14243-bib-0022] Lualdi, M. , Alberio, T. , & Fasano, M. (2020). Proteostasis and Proteotoxicity in the network medicine era. International Journal of Molecular Sciences, 21, E6405. 10.3390/ijms21176405 PMC7503343 32899160

[acel14243-bib-0023] Martinez‐Espinosa, P. L. , Wu, J. , Yang, C. , Gonzalez‐Perez, V. , Zhou, H. , Liang, H. , Xia, X.‐M. , & Lingle, C. J. (2015). Knockout of Slo2.2 enhances itch, abolishes KNa current, and increases action potential firing frequency in DRG neurons. eLife, 4, e10013. 26559620 10.7554/eLife.10013 PMC4641468

[acel14243-bib-0024] McDaid, D. , Park, A.‐L. , & Chadha, S. (2021). Estimating the global costs of hearing loss. International Journal of Audiology, 60, 162–170. 33590787 10.1080/14992027.2021.1883197

[acel14243-bib-0025] McFadden, S. L. , Ding, D. , Burkard, R. F. , Jiang, H. , Reaume, A. G. , Flood, D. G. , & Salvi, R. J. (1999). Cu/Zn SOD deficiency potentiates hearing loss and cochlear pathology in aged 129, CD‐1 mice. The Journal of Comparative Neurology, 413, 101–112. 10464373

[acel14243-bib-0026] Mulry, E. , & Parham, K. (2020). Inner ear proteins as potential biomarkers. Otology & Neurotology, 41, 145–152. 31789810 10.1097/MAO.0000000000002466

[acel14243-bib-0027] Ohlemiller, K. K. , McFadden, S. L. , Ding, D. L. , Flood, D. G. , Reaume, A. G. , Hoffman, E. K. , Scott, R. W. , Wright, J. S. , Putcha, G. V. , & Salvi, R. J. (1999). Targeted deletion of the cytosolic Cu/Zn‐superoxide dismutase gene (Sod1) increases susceptibility to noise‐induced hearing loss. Audiology & Neuro‐Otology, 4, 237–246. 10436316 10.1159/000013847

[acel14243-bib-0028] Olzmann, J. A. , Li, L. , & Chin, L. S. (2008). Aggresome formation and neurodegenerative diseases: Therapeutic implications. Current Medicinal Chemistry, 15, 47–60. 18220762 10.2174/092986708783330692 PMC4403008

[acel14243-bib-0029] Parthasarathy, A. , Hancock, K. E. , Bennett, K. , DeGruttola, V. , & Polley, D. B. (2020). Bottom‐up and top‐down neural signatures of disordered multi‐talker speech perception in adults with normal hearing. eLife, 9, e51419. 31961322 10.7554/eLife.51419 PMC6974362

[acel14243-bib-0030] Peixoto Pinheiro, B. , Vona, B. , Löwenheim, H. , Rüttiger, L. , Knipper, M. , & Adel, Y. (2021). Age‐related hearing loss pertaining to potassium ion channels in the cochlea and auditory pathway. Pflügers Archiv, 473, 823–840. 33336302 10.1007/s00424-020-02496-w PMC8076138

[acel14243-bib-0031] Pouyo, R. , Chung, K. , Delacroix, L. , & Malgrange, B. (2021). The ubiquitin‐proteasome system in normal hearing and deafness. Hearing Research, 426, 108366. 34645583 10.1016/j.heares.2021.108366

[acel14243-bib-0046] Pras, A. , & Nollen, E. A. A. (2021). Regulation of age‐related protein toxicity. Frontiers in Cell and Developmental Biology, 9, 637084. 33748125 10.3389/fcell.2021.637084 PMC7973223

[acel14243-bib-0032] Reijntjes, D. O. J. , Breitzler, J. L. , Persic, D. , & Pyott, S. J. (2021). Preparation of the intact rodent organ of Corti for RNAscope and immunolabeling, confocal microscopy, and quantitative analysis. STAR Protocols, 2, 100544. 34195667 10.1016/j.xpro.2021.100544 PMC8233256

[acel14243-bib-0033] Reijntjes, D. O. J. , Lee, J. H. , Park, S. , Schubert, N. M. A. , van Tuinen, M. , Vijayakumar, S. , Jones, T. A. , Jones, S. M. , Gratton, M. A. , Xia, X.‐M. , Yamoah, E. N. , & Pyott, S. J. (2019). Sodium‐activated potassium channels shape peripheral auditory function and activity of the primary auditory neurons in mice. Scientific Reports, 9, 2573. 30796290 10.1038/s41598-019-39119-z PMC6384918

[acel14243-bib-0034] Reijntjes, D. O. J. , Schubert, N. M. A. , Pietrus‐Rajman, A. , van Dijk, P. , & Pyott, S. J. (2018). Changes in spontaneous movement in response to silent gaps are not robust enough to indicate the perception of tinnitus in mice. PLoS One, 13, e0202882. 30157212 10.1371/journal.pone.0202882 PMC6114799

[acel14243-bib-0035] Reimand, J. , Isserlin, R. , Voisin, V. , Kucera, M. , Tannus‐Lopes, C. , Rostamianfar, A. , Wadi, L. , Meyer, M. , Wong, J. , Xu, C. , Merico, D. , & Bader, G. D. (2019). Pathway enrichment analysis and visualization of omics data using g:Profiler, GSEA, Cytoscape and EnrichmentMap. Nature Protocols, 14, 482–517. 30664679 10.1038/s41596-018-0103-9 PMC6607905

[acel14243-bib-0036] Ruz, C. , Alcantud, J. L. , Vives Montero, F. , Duran, R. , & Bandres‐Ciga, S. (2020). Proteotoxicity and neurodegenerative diseases. International Journal of Molecular Sciences, 21, 5646. 32781742 10.3390/ijms21165646 PMC7460676

[acel14243-bib-0037] Schubert, N. M. A. , van Tuinen, M. , & Pyott, S. J. (2022). Transcriptome‐guided identification of drugs for repurposing to treat age‐related hearing loss. Biomolecules, 12, 498. 35454087 10.3390/biom12040498 PMC9028743

[acel14243-bib-0038] Shen, J. , Scheffer, D. I. , Kwan, K. Y. , & Corey, D. P. (2015). SHIELD: An integrative gene expression database for inner ear research. Database: The Journal of Biological Databases and Curation, 2015, bav071. 26209310 10.1093/database/bav071 PMC4513695

[acel14243-bib-0039] Shrestha, B. R. , Chia, C. , Wu, L. , Kujawa, S. G. , Liberman, M. C. , & Goodrich, L. V. (2018). Sensory neuron diversity in the inner ear is shaped by activity. Cell, 174, 1229–1246.e17. 30078709 10.1016/j.cell.2018.07.007 PMC6150604

[acel14243-bib-0040] Spankovich, C. , Gonzalez, V. B. , Su, D. , & Bishop, C. E. (2018). Self reported hearing difficulty, tinnitus, and normal audiometric thresholds, the national health and nutrition examination survey 1999–2002. Hearing Research, 358, 30–36. 29254853 10.1016/j.heares.2017.12.001

[acel14243-bib-0041] Tremblay, K. L. , Pinto, A. , Fischer, M. E. , Klein, B. E. K. , Klein, R. , Levy, S. , Tweed, T. S. , & Cruickshanks, K. J. (2015). Self‐reported hearing difficulties among adults with normal audiograms: The beaver dam offspring study. Ear and Hearing, 36, e290–e299. 26164105 10.1097/AUD.0000000000000195 PMC4824300

[acel14243-bib-0042] Wan, G. , & Corfas, G. (2017). Transient auditory nerve demyelination as a new mechanism for hidden hearing loss. Nature Communications, 8, 14487. 10.1038/ncomms14487 PMC5321746 28211470

[acel14243-bib-0043] Wilson, D. M. , Cookson, M. R. , Van Den Bosch, L. , Zetterberg, H. , Holtzman, D. M. , & Dewachter, I. (2023). Hallmarks of neurodegenerative diseases. Cell, 186, 693–714. 36803602 10.1016/j.cell.2022.12.032

[acel14243-bib-0044] Yu, W. , Zong, S. , Du, P. , Zhou, P. , Li, H. , Wang, E. , & Xiao, H. (2021). Role of the Stria Vascularis in the pathogenesis of sensorineural hearing loss: A narrative review. Frontiers in Neuroscience, 15, 774585. 34867173 10.3389/fnins.2021.774585 PMC8640081

[acel14243-bib-0045] Zheng, Q. Y. , Johnson, K. R. , & Erway, L. C. (1999). Assessment of hearing in 80 inbred strains of mice by ABR threshold analyses. Hearing Research, 130, 94–107. 10320101 10.1016/s0378-5955(99)00003-9 PMC2855304

