# Supplementary material for: Pathophysiological processes underlying hidden hearing loss revealed in Kcnt1/2 double knockout mice
Source: Aging Cell. 2024 Jul 24;23(9):e14243. doi: 10.1111/acel.14243 (PMC11488318; doi:10.1111/acel.14243)
Supplement: Supplementary file 1 — Figure S1. [file ACEL-23-e14243-s004.docx]

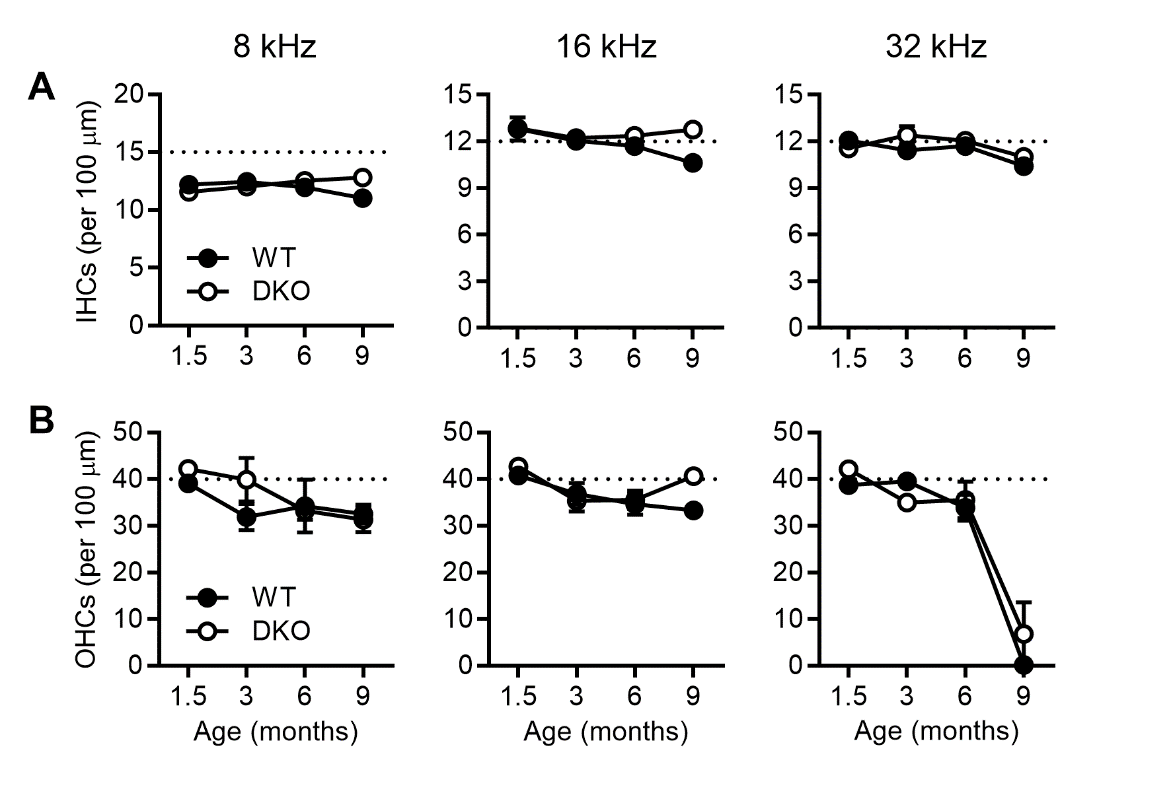


**Supplementary Figure S1. DKO and WT mice show comparable age-related loss of inner and outer hair cells.** Inner hair cell density (A, IHC per 100 µm) and outer hair cell density (B, OHC per 100 per 100 µm) are shown as a function of age for WT (filled circles) and DKO mice (open circles) for the indicated tonotopic regions. At each age examined, no significant differences were observed between genotypes.
